# Assessment of prostate cancer aggressiveness through the combined analysis of prostate MRI and 2.5D deep learning models

**DOI:** 10.3389/fonc.2025.1539537

**Published:** 2025-06-30

**Authors:** Yalei Wang, Yuqing Xin, Baoqi Zhang, Fuqiang Pan, Xu Li, Manman Zhang, Yushan Yuan, Lei Zhang, Peiqi Ma, Bo Guan, Yang Zhang

**Affiliations:** ^1^ Department of Radiology, Fuyang People’s Hospital of Anhui Medical University, Fuyang, China; ^2^ Department of Radiology, Fuyang People’s Hospital of Bengbu Medical University, Fuyang, China; ^3^ Department of Radiology, Fuyang People’s Hospital, Fuyang, China; ^4^ Department of Urology, Fuyang People’s Hospital, Fuyang, China

**Keywords:** prostate cancer, aggressiveness, MRI, radiomics, deep learning, nomogram

## Abstract

**Objective:**

Prostate cancer is prevalent among older men. Although this malignancy has a relatively low mortality rate, its aggressiveness is critical in determining patient prognosis and treatment options. This study therefore aimed to evaluate the effectiveness of a 2.5D deep learning model based on prostate MRI to assess prostate cancer aggressiveness.

**Materials and methods:**

This study included 335 patients with pathologically-confirmed prostate cancer from a tertiary medical center between January 2022 and December 2023. Of these, 266 cases were classified as aggressive and 69 as non-aggressive, using a Gleason score ≥7 as the cutoff. The subjects were automatically divided into a test set and validation set in a 7:3 ratio. Before pathological biopsy, all patients underwent biparametric MRI, including T2-weighted imaging, diffusion-weighted imaging, and apparent diffusion coefficient scans. Two radiologists, blinded to pathology results, segmented the lesions using ITK-SNAP software, extracting the minimal bounding rectangle of the largest ROI layer, along with the corresponding ROIs from adjacent layers above and below it. Subsequently, radiomic features were extracted using pyradiomics tool, while deep learning features from each cross-section were derived using the Inception_v3 neural network. To ensure consistency in feature extraction, intraclass correlation coefficient (ICC) analysis was performed on features extracted by radiologists, followed by feature normalization using the mean and standard deviation of the training set. Highly correlated features were removed using t-tests and Pearson correlation tests, and redundant features were ultimately screened with least absolute shrinkage and selection operator (Lasso). Models were constructed using the LightGBM algorithm: a radiomic feature model, a deep learning feature model, and a combined model integrating radiomic and deep learning features. Further, a clinical feature model (Clinic-LightGBM) was constructed using LightGBM to include clinical information. The optimal feature model was then combined with Clinic-LightGBM to establish a nomogram. The Grad-CAM technique was employed to explain the deep learning feature extraction process, supported by tree model visualization techniques to illustrate the decision-making process of the LightGBM model. Model classification performance in the test set was evaluated using the area under the receiver operating characteristic curve (AUC).

**Results:**

In the test set, the nomogram demonstrated the highest predictive ability for prostate cancer aggressiveness (AUC = 0.919, 95% CI: 0.8107–1.0000), with a sensitivity of 0.966 and specificity of 0.833. The DLR-LightGBM model (AUC = 0.872) outperformed the DL-LightGBM (AUC = 0.818) and Rad-LightGBM (AUC = 0.758) models, indicating the benefit of combining deep learning and radiomic features.

**Conclusion:**

Our 2.5D deep learning model based on prostate MRI showed efficacy in identifying clinically significant prostate cancer, providing valuable references for clinical treatment and enhancing patient net benefit.

## 1 Introduction

Prostate cancer is the second most common malignancy among men worldwide (1). As an age-related tumor, it has a high incidence rate, but relatively low mortality (2). Low-grade prostate cancer typically grows slowly, with minimal risk of dissemination, allowing for active surveillance or localized treatment. However, high-grade prostate cancer generally requires more aggressive and diverse clinical management to control disease progression, prolong survival, and improve patient quality of life (3).

Traditional imaging diagnosis of prostate lesions and aggressiveness assessment relies predominantly on multiparametric prostate MRI and the interpretation of the Prostate Imaging–Reporting and Data System (PI-RADS) v2.1 (4)​. This includes assessments of lesion size, signal intensity, enhancement patterns, and invasion into the surrounding tissues (5). However, the complex growth patterns of prostate cancer often leads to inter- and intra-observer variability in classification (6). The Gleason score (7), obtained via transrectal ultrasound or MRI-guided biopsy, is a key indicator of prostate cancer aggressiveness. However, this system has a relatively low sensitivity (8), and thus inflicts economic and psychological burdens to patients. As such, there is an urgent need for a non-invasive, rapid, and effective imaging tool to assess prostate cancer aggressiveness.

With advances in artificial intelligence, deep learning has been widely applied in the field of medical image analysis (9)​​. Deep learning, by mimicking the connections of human neurons, can automatically learn and extract high-level image features from large-scale imaging data, which are often undetectable to the human eye. These features are applied to risk stratification and treatment planning, thereby significantly enhancing diagnostic accuracy and efficiency (10). In previous studies, 2D deep learning and radiomics have been widely applied (11–13). However, their limited ability to capture 3D spatial information poses challenges in analyzing complex tumor structures. Conversely, Unlike previous studies relying on 2D deep learning or radiomics alone, our approach introduces a 2.5D deep learning framework that partially captures 3D spatial information while maintaining computational efficiency. Additionally, we integrate radiomic features, deep learning, and clinical variables into a nomogram, offering a more comprehensive tool for aggressiveness assessment. This study aimed to integrate multiparametric prostate MRI with advanced machine learning techniques to develop a novel 2.5D deep learning model, to enhance accuracy in prostate cancer diagnosis and aggressiveness assessment to support clinical decision-making.

## 2 Materials and methods

### 2.1 Patients

**Figure 1:**
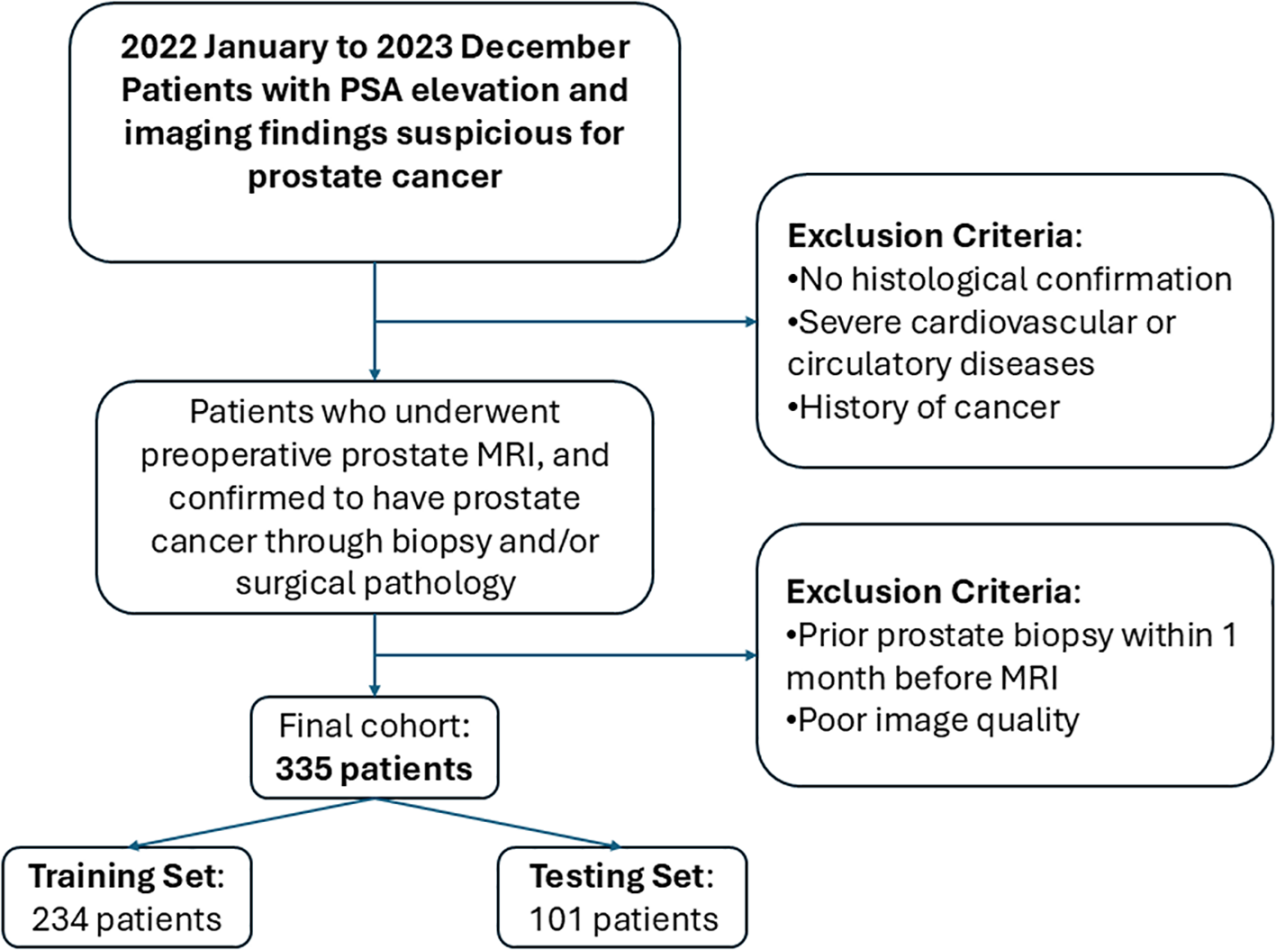
Flowchart showing patient inclusion and exclusion.

This retrospective study included prostate cancer patients diagnosed at a tertiary medical center from January 2022 to December 2023. All patients underwent preoperative multiparametric MRI, with prostate cancer confirmed through biopsy and postoperative pathological examination. The inclusion criteria were as follows: patients who underwent preoperative multiparametric prostate magnetic resonance imaging (MRI) with confirmed pathological diagnosis of prostate cancer. The exclusion criteria were as follows: lack of histological confirmation, severe cardiovascular or circulatory disease, a prior history of cancer, poor image quality, or biopsy within one month prior to MRI. Ultimately, the MRI and clinical data from 335 patients were included, with patients categorized as high-aggressiveness (266 cases) or low-aggressiveness (69 cases) based on a Gleason score threshold of ≥7. The data were split into training and test sets in a 7:3 ratio, with the test set used for model evaluation to ensure validity and reliability. The patient inclusion and exclusion process is presented in **Figure 1**.

### 2.2 Image acquisition

All patients underwent biparametric prostate MRI prior to pathological biopsy, including T2-weighted imaging (T2WI), diffusion-weighted imaging (DWI), and apparent diffusion coefficient (ADC) measurements. MRI scans were performed using a Philips 3.0T high-field strength MRI scanner to ensure high-resolution imaging. All examinations followed the PI-RADS v2.0 or v2.1 technical guidelines. **Table 1** provides the details of the MRI acquisition parameters.

**Table 1:** Overview of the scanning parameters and sequences.

Sequence	Plane	TR (ms)	TE (ms)	Slice thickness (mm)	Slice gap (mm)	FOV (mm)	Matrix	b-value (s/mm²)	Time resolution (s)	Other parameters
T2WI	Axial	3000–4000	80–120	3	0.3	180–220	320 x 320	N/A	N/A	- TSE factor: 16–20
										- NSA: 1–2
T2WI	Sagittal	3000–4000	80–120	3	0.3	180–220	320 x 320	N/A	N/A	- TSE factor: 16-20
										- NSA: 1–2
T2WI	Coronal	3000–4000	80–120	3	0.3	180–220	320 x 320	N/A	N/A	- TSE factor: 16-20
										- NSA: 1–2
DWI	Axial	3000–4000	60–90	3	0.3	180–220	128 x 128	0, 50–100, 800–1000	N/A	- High b-value (1400–2000) calculated

N/A, Not Applicable.

### 2.3 Prostate pathology

Pathology results were obtained from pathology reports, with two experienced uropathologists independently reviewing all slides and performing grading according to the Gleason scoring system. In cases of discrepancy, a third pathologist was consulted to achieve consensus. In the present study, Gleason scores ≥7 were defined as aggressive prostate cancer, while scores ≤6 were classified as non-aggressive prostate cancer.

### 2.4 Clinical data

Basic clinical data were collected, including age, prostate-specific antigen (PSA) levels, and Prostate Imaging Reporting & Data System (PI-RADS) scores. All data were extracted from the patients’ medical records, with data on PSA levels taken from the most recent test before MRI, and PI-RADS scores from radiology reports. Data were entered into standardized tables and double-checked for accuracy. Chi-square tests were used for categorical variables. Baseline statistics were performed on all clinical characteristics to ensure group consistency.

### 2.5 MRI data preprocessing

All MRI images were exported from the Picture Archiving and Communication System (PACS) in DICOM format. Following conversion to the NII format, all imaging data were resampled to a fixed resolution with voxel spacing standardized to 1mm × 1mm × 1mm. Next, the CT Hounsfield Units (HU) were normalized to a range of -120 to 180, corresponding to a window width of 300 and window level of 30. This standardization enhances robustness in medical image analysis.

### 2.6 Radiomics workflows

#### 2.6.1 Image segmentation and cropping

**Figure 2:**
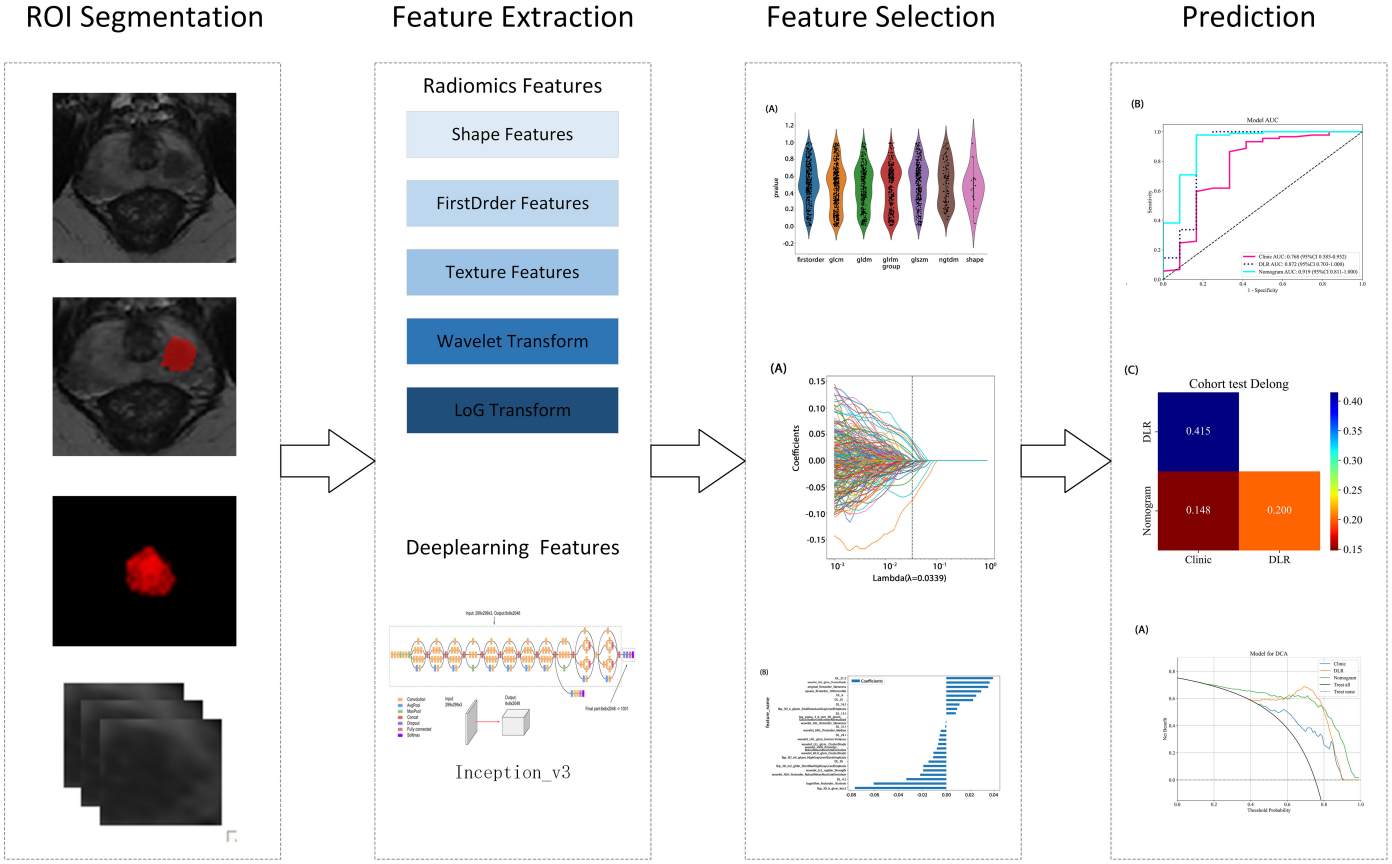
Overview of the study workflow.

Two radiologists, blinded to the pathology results, used ITK-SNAP (version 3.8.0, http://www.itksnap.org) to perform layer-by-layer segmentation of the region of interest (ROI) along the lesion edges on axial T2WI, combined with DWI sequences and ADC images. For patients with multiple lesions, only the lesion with the highest PI-RADS 2.1 score was segmented. Next, the minimum bounding rectangle of the largest ROI slice and two adjacent slices above and below were cropped. Missing slices were further filled with symmetric counterparts. To ensure consistency in feature extraction, an inter-rater consistency check was first conducted. After one month, the radiologists re-segmented randomly selected images from 20 patients to assess the intra-rater consistency. Deep learning features with an intraclass correlation coefficient (ICC) > 0.8 were selectively retained. This approach ensured the stability and reliability of segmentation results, providing a robust data foundation for further analysis and model development. **Figure 2** presents the detailed workflow of the study.

#### 2.6.2 Feature extraction and filtering

**Figure 3:**
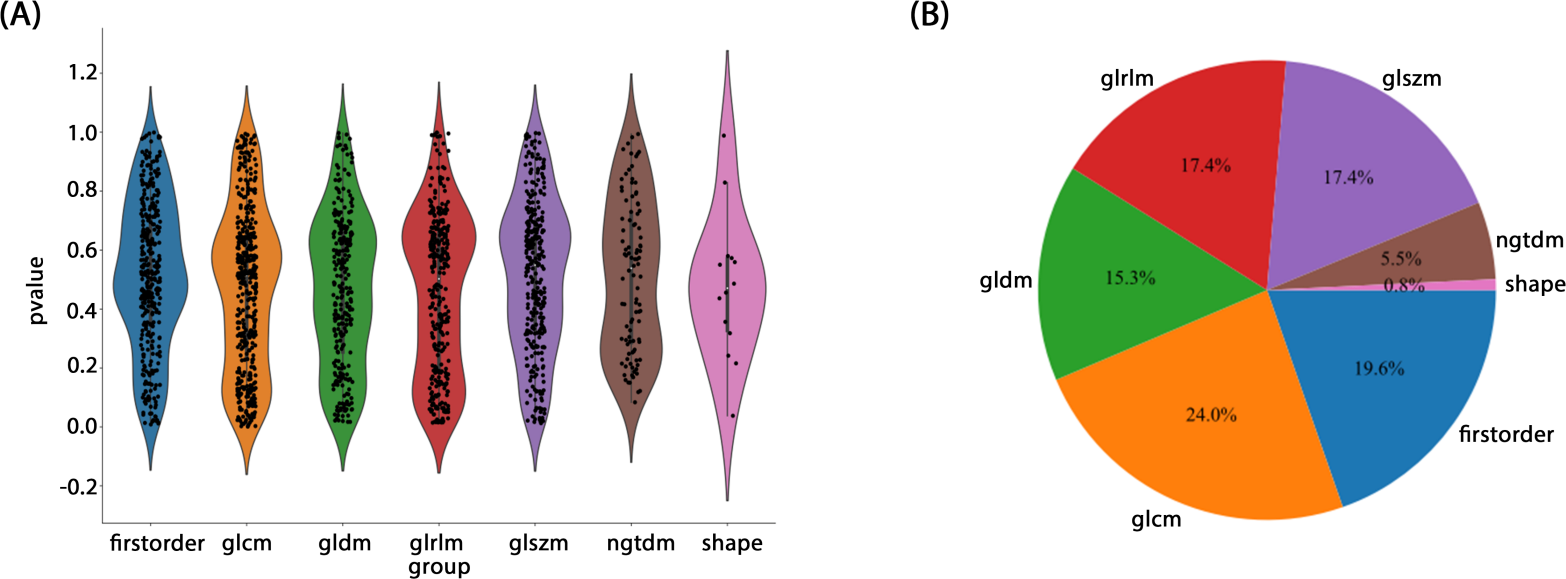
**(A, B)** Ratio of the different radiomics features.

Radiomic features were extracted from segmented ROIs using pyradiomics (version 3.0.1), adhering to the rigorous standards set by the Imaging Biomarker Standardization Initiative (IBSI). A total of 1,834 features were extracted and categorized into four groups: first-order statistical, shape, texture, and filter-based high-order features. Each feature category captures distinct tumor attributes across various dimensions: first-order features describe the basic pixel intensity statistics, shape features reflect ROI geometry, texture features quantify local intensity patterns and relationships in grayscale images, and high-order features uncover complex spatial information via filters such as wavelet transforms. **Figure 3** presents the specific feature distribution.

First, a grid search was conducted on the training set data to determine the optimal deep learning network, Inception_v3, for feature extraction. Then, the 2D deep learning features were extracted from each MRI slice. Subsequently, adjacent slice information was integrated through feature fusion to construct a partially 3D spatial representation as 2.5D deep learning features. This method retained the computational efficiency and data requirements of a 2D model, while enhancing the ability to capture tumor spatial relationships through multilayer image integration. This efficient and accurate feature extraction method offers an innovative approach for prostate cancer imaging analysis.

All features were normalized using the mean and standard deviation of the training set to ensure consistent feature scaling, thereby avoiding biases during model training. In the statistical analysis, a t-test was initially applied to identify the features significantly associated with prostate cancer aggressiveness. A significance threshold of p < 0.05 was applied, retaining only features meeting this criterion for further analysis. Next, Pearson’s correlation tests were applied to assess feature correlations, with a threshold of 0.9 to remove highly correlated features and avoid multicollinearity effects in the model. Finally, Lasso regression with L1 regularization was applied for redundant feature selection, effectively identifying the most predictive features and automatically removing redundant ones. To ensure robust feature selection, the Lasso regularization parameter (λ) was determined through 10-fold cross validation, selecting the optimal parameter to maintain the model’s generalizability. This feature selection process strictly followed statistical and machine learning optimization principles, aiming to improve predictive performance, while minimizing the risk of overfitting.

#### 2.6.3 Model construction and evaluation

In this study, all of the enrolled patients were randomly assigned to the training and testing sets in a 7:3 ratio, thereby ensuring the independence of model training and validation. The study extracted radiomic and 2.5D deep learning features based on T2WI images. Following rigorous selection, models were constructed using the LightGBM algorithm: one based on radiomic features (Rad-LightGBM), one on deep learning features (DL-LightGBM), and a combined model integrating both feature types (DLR-LightGBM). Additionally, a clinical feature model (Clinic-LightGBM) was constructed using the LightGBM algorithm to incorporate clinical data in the analysis. Finally, the top-performing feature model was combined with Clinic-LightGBM to develop a nomogram integrating multi-source features and clinical variables to predict individual patient risk probabilities, to provide a quantitative tool to enhance individualized accuracy in prostate cancer diagnosis and support clinical decision-making.

For model evaluation, the area under the receiver operating characteristic (ROC) curve (AUC) was first calculated to quantify the model’s overall classification performance. Additionally, the model’s accuracy, sensitivity, and specificity were calculated to comprehensively assess its performance on the test set. To further validate the model’s clinical applicability, decision curve analysis (DCA) was conducted to measure the net benefit across different thresholds, to assess the model’s potential contribution to clinical decision-making. To compare the performance differences between models (deep learning model, clinical feature model, and combined model), DeLong’s test was applied to statistically assess the significance of AUC differences, thereby ensuring reliability in performance comparisons.

### 2.7 Statistics

All statistical analyses were performed using Python 3.7.12, with the LightGBM machine learning algorithm implemented via Scikit-learn version 1.0.2. The Shapiro-Wilk test was subsequently applied to assess the normality of clinical features in binary variables, and depending on the normality distribution, either the t-test or Mann-Whitney U test was used for significance assessment. Chi-square tests were applied for categorical variables. Finally, univariate regression analysis was conducted on all clinical features to identify those with p < 0.05.

### 2.8 Grad-CAM

Gradient-weighted Class Activation Mapping (Grad-CAM) was employed in this study to interpret the feature extraction process of the deep learning model. Grad-CAM is a widely used technique that generates heatmaps, highlighting regions of input images critical to model predictions, and thereby providing a visual interpretation of deep learning models. Specifically, we applied Grad-CAM visualization to the third-to-last layer, the final convolutional layer of the Inception_v3 model, to identify spatial regions associated with malignancy or aggressiveness. The heatmaps visually reveal the deep learning model’s decision-making process, addressing the “black-box” issue commonly associated with deep networks. By overlaying the Grad-CAM heatmaps onto the original MRI images, we were able clearly observe the regions with the greatest influence on model output, thereby allowing us to evaluate if these areas align with known pathological features of prostate cancer.

### 2.9 Tree model visualization

In this study, tree model visualization techniques were applied to interpret the model constructed using the LightGBM algorithm, an efficient, decision tree-based gradient boosting algorithm with strong capabilities for handling large-scale and sparse datasets. Tree model visualization focuses on two aspects: displaying feature importance and tracing individual sample decision paths. Feature importance reflects the relative contribution of each input variable to the model’s predictions, thereby aiding in the identification of features critical to the model’s decision-making. Additionally, the visualization of decision paths reveals how the model incrementally reaches classifications or predictions. For example, for one specific patient, we are able to trace the model’s decisions at each tree node based on specific feature values, thereby culminating in the prediction outcome. This decision path visualization provides clinicians with intuitive decision support, and offers a more transparent model interpretation, helping to build user trust in the model’s predictions.

## 3 Results

### 3.1 Patient characteristics

Data from 335 eligible patients were included in the study, comprising 266 and 69 cases of aggressive and non-aggressive prostate cancer, respectively. Statistical tests showed no significant differences in age, PSA levels, maximum tumor length, or PI-RADS 2.1 scores between groups in either the training or test sets (P > 0.05), indicating unbiased grouping. Clinical baseline statistics across groups are presented in **Table 2**. In this study, we conducted a comprehensive univariate analysis of all clinical features, calculating each feature’s odds ratio (OR) and associated p-value. Variables with clinical significance (p < 0.05) were included in the subsequent clinical feature model.

**Table 2:** Clinical baseline characteristics of prostate cancer patients in different groups.

Feature_name	Label=all	Label=test	Label=train	P-value
PSA	56.26 ± 119.20	64.39 ± 118.80	52.75 ± 119.46	0.053
Age	70.03 ± 7.90	71.06 ± 7.62	69.59 ± 8.00	0.075
Tumor length	22.88 ± 13.80	22.74 ± 12.53	22.94 ± 14.34	0.736
pi_rads				0.626
1	10 (2.99)	1 (0.99)	9 (3.85)	
2	40 (11.94)	11 (10.89)	29 (12.39)	
3	88 (26.27)	28 (27.72)	60 (25.64)	
4	62 (18.51)	21 (20.79)	41 (17.52)	
5	135 (40.30)	40 (39.60)	95 (40.60)	

### 3.2 Feature extraction and filtering

**Figure 4:**
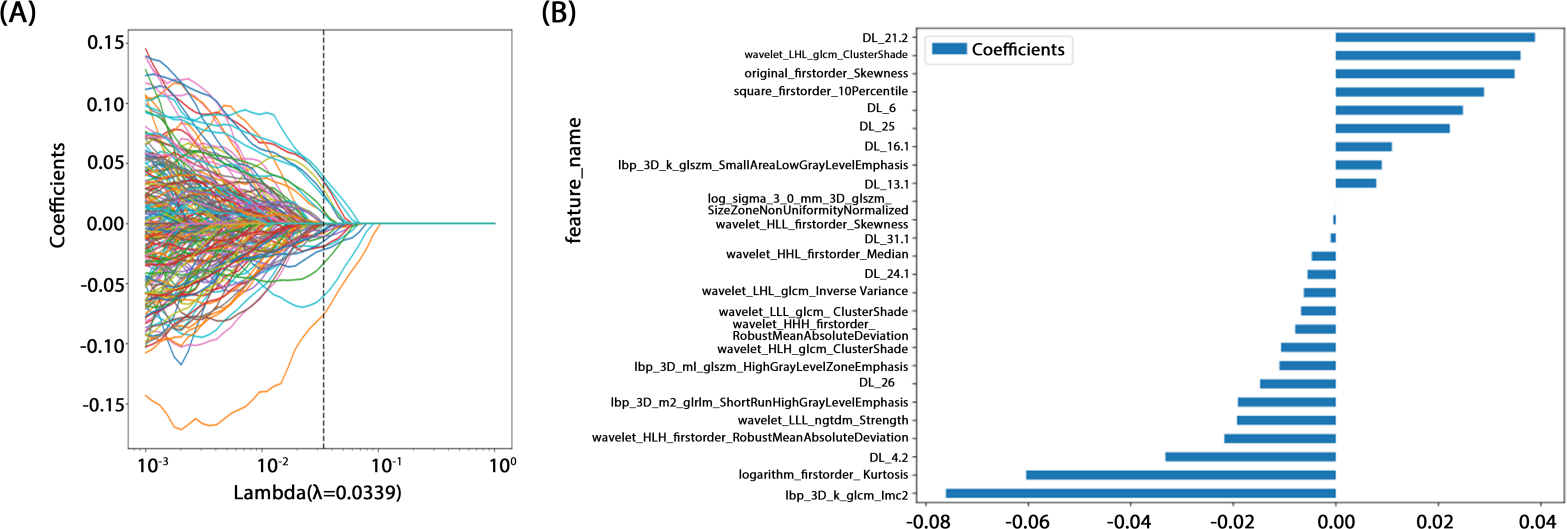
**(A)** Coefficients of 10-fold cross validation. **(B)** Histogram of the Rad-score based on the selected features.

A rigorous feature selection process was applied to the extract core features from different types of initial feature sets for model construction. Specifically, 11 features were selected from amongst 1834 radiomic features, 41 from 6144 deep learning features (generated by combining 2048-dimensional features across three slices), and 26 from the combined feature set. Additionally, four clinical features (PSA level, maximum tumor length, age, and PI-RADS 2.1 score) were included. Multistep statistical analyses and machine learning methods ensured that the selected features were significant in differentiating prostate cancer aggressiveness. By combining statistical tests and dimensionality reduction algorithms, we were able to remove feature redundancy, and minimized the risk of overfitting, thereby improving model generalizability and predictive stability. Through this optimized feature selection strategy, we were able to develop a model with high predictive performance and interpretability, thereby providing a scientific basis to assess prostate cancer aggressiveness. The feature selection process is illustrated in **Figure 4**.

### 3.3 Model construction and evaluation

In this study, all of the constructed models demonstrated high classification performance on the training set (AUC > 0.8). In the test set, the three models combining 2.5D deep learning and radiomic features outperformed the clinical feature-only model (AUC = 0.757, 95% CI: 0.5726 - 0.9414). Among these, two models incorporating 2.5D deep learning features performed better than the radiomics-only model. The DLR-LightGBM model achieved the highest classification performance, with an AUC of 0.872 (95% CI: 0.7030 - 1.0000), significantly outperforming both the DL-LightGBM (AUC = 0.818, 95% CI: 0.6659 - 0.9708) and the Rad-LightGBM (AUC = 0.758, 95% CI: 0.5518 - 0.9641) models. These results indicate that combining deep learning and radiomic features can significantly improve the predictive power of models in assessing prostate cancer aggressiveness, thereby allowing a more comprehensive evaluation. Furthermore, the DLR-LightGBM model demonstrated exceptional sensitivity and specificity on the test set, with values of 0.966 and 0.833, respectively, with specificity surpassing that of other models, underscoring its value in evaluating prostate cancer aggressiveness.

**Figure 5:**
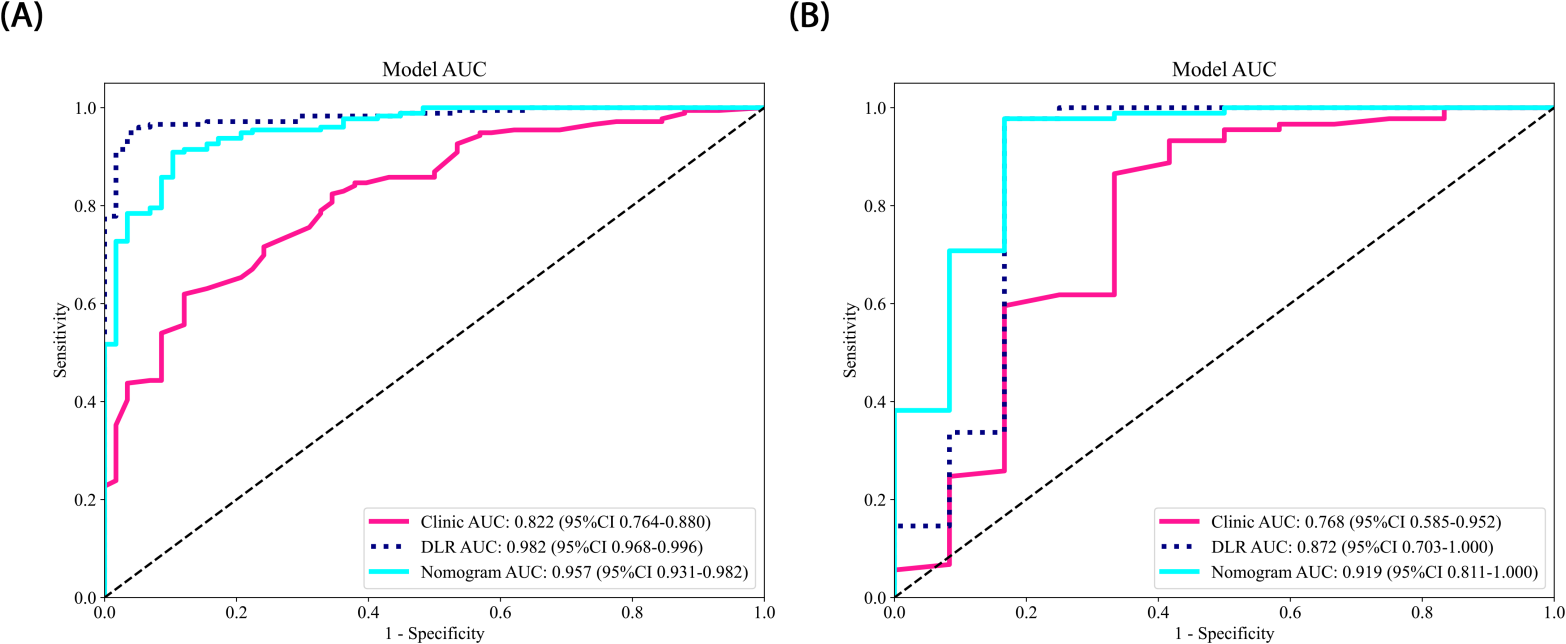
**(A, B)** ROC Curves of the different Models in each cohort.

**Figure 6:**
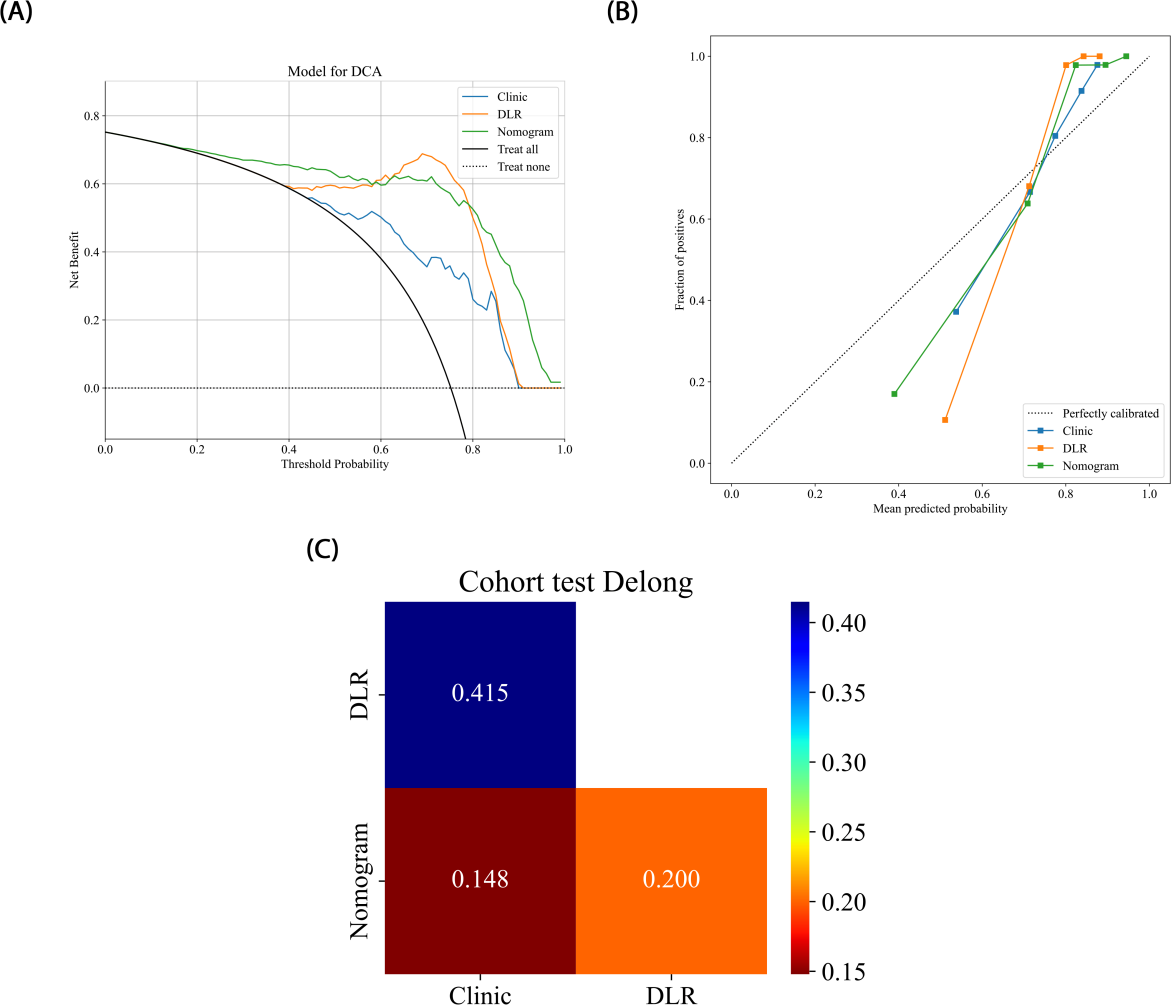
**(A)** Different signatures’ decision curves on the test cohort. **(B)** Different signatures’ calibration curve on the test cohort. **(C)** Delong scores of different signature.

The nomogram, a comprehensive model integrating imaging and clinical features, also demonstrated strong predictive performance, achieving an AUC of 0.919 (95% CI: 0.8107 - 1.0000), with a sensitivity of 0.966 and specificity of 0.833 (**Table 3**) (**Figure 5**). The results of the DeLong test suggest that the nomogram’s classification performance on the test set was almost significantly superior to the DLR-LightGBM and Clinic-LightGBM models, indicating that the nomogram holds substantial clinical utility in predicting prostate cancer aggressiveness, thereby offering valuable guidance in clinical decision-making. DCA further showed that the nomogram maintained a high clinical net benefit across nearly the entire range of risk thresholds. The Hosmer-Lemeshow (HL) test indicated strong agreement between predicted and observed outcomes for the nomogram (**Figure 6**). These findings highlight that the nomogram not only outperforms other models in terms of accuracy and calibration, but also has significant potential for application in managing and decision-making for high-risk prostate cancer patients.

**Table 3:** Prediction performance of the different models.

Signature	AUC	95% CI	Sensitivity	Specificity	PPV	NPV	Cohort
DL_LightGBM	0.995	0.9889 - 1.0000	0.989	0.948	0.983	0.965	Train
DLR_LightGBM	0.982	0.9680 - 0.9962	0.943	0.966	0.988	0.848	Train
Rad_LightGBM	0.899	0.8553 - 0.9421	0.864	0.776	0.921	0.652	Train
Clinic_LightGBM	0.822	0.7637 - 0.8801	0.602	0.879	0.938	0.421	Train
Nomogram	0.957	0.9308 - 0.9822	0.903	0.897	0.964	0.754	Train
DL_LightGBM	0.818	0.6659 - 0.9708	0.989	0.583	0.946	0.875	Test
DLR_LightGBM	0.872	0.7030 - 1.0000	0.966	0.833	0.977	0.769	Test
Rad_LightGBM	0.758	0.5518 - 0.9641	0.798	0.75	0.959	0.333	Test
Clinic_LightGBM	0.768	0.5845 - 0.9520	0.854	0.667	0.949	0.381	Test
Nomogram	0.919	0.8107 - 1.0000	0.966	0.833	0.977	0.769	Test

### 3.4 Grad-CAM evaluation

**Figure 7:**
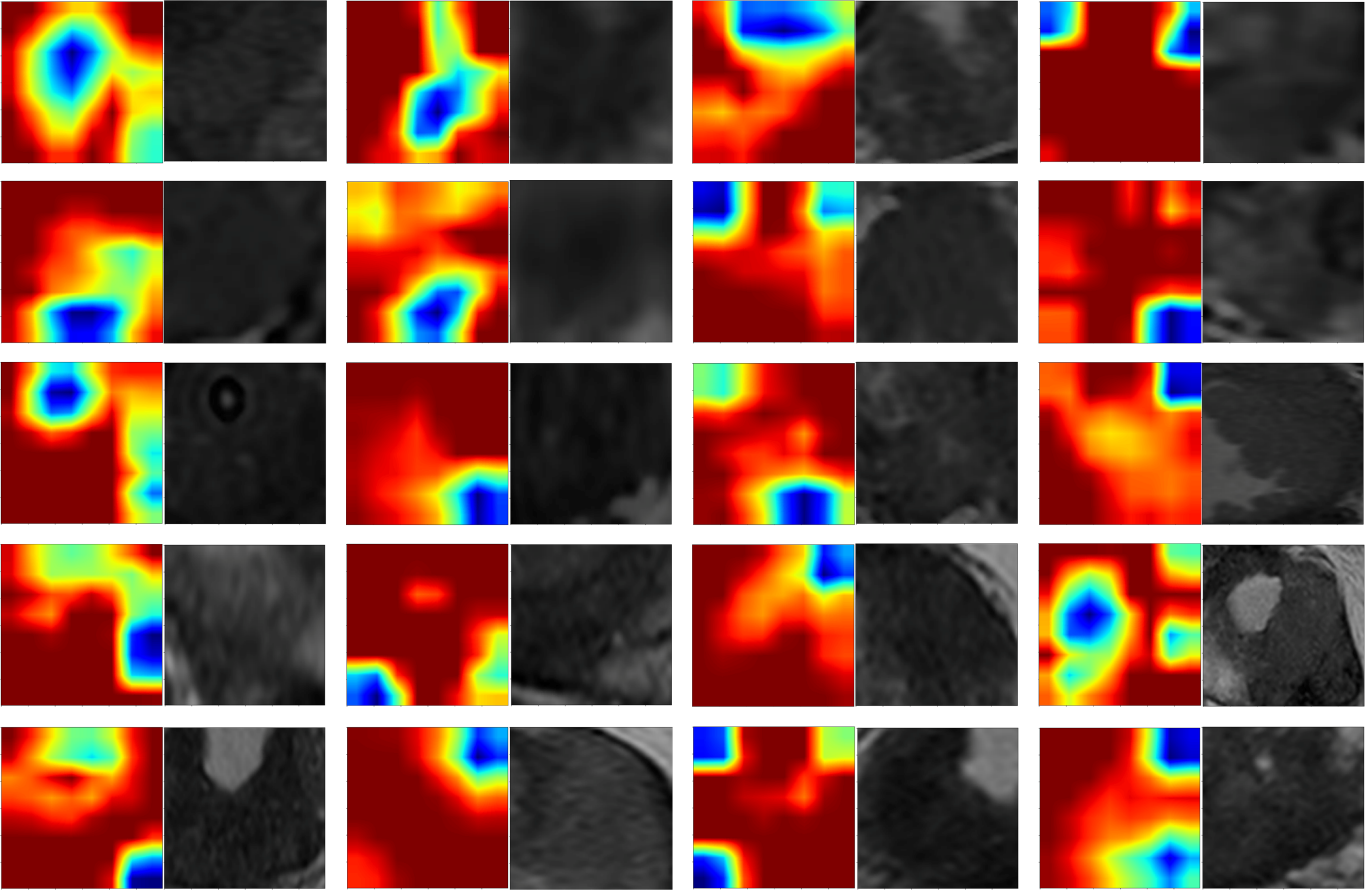
Grad-CAM heatmap.

In this study, Grad-CAM visualization was applied to images from 101 test-set patients to evaluate the activation of the deep learning model in prostate cancer lesion areas. Additionally, pathology specimens from 20 randomly selected patients were retrospectively analyzed. Results showed that, highlighted areas in the heatmaps significantly overlapped with the segmented lesion regions in all 20 samples, demonstrating the model’s sensitivity and accuracy in detecting tumor areas. Among these 20 samples, 19 displayed heatmap activation areas that matched prostate cancer-diagnosed regions in pathology reports, with regions of high activation correlating to blurred or absent gland structures and abnormal cellular arrangement in pathology specimens. This result indicates that Grad-CAM-generated heatmaps effectively highlight key regions of model focus, aligning with actual lesion locations, and enhancing the model’s interpretability. This visualization analysis further validates the model’s effectiveness in identifying prostate cancer in imaging, thereby providing valuable insights for clinical diagnosis and future research (**Figure 7**).

### 3.5 LightGBM tree model visualization

**Figure 8:**
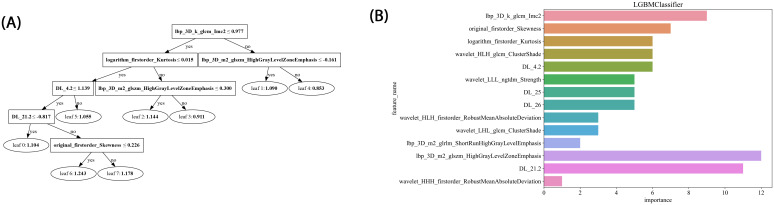
**(A)** Histogram of the Rad-score based on the selected features. **(B)** LightGBM tree model decision diagram.

In this study, LightGBM tree model visualization techniques were employed to display the influence of different features in the model’s decision-making process. By analyzing the decision paths in the tree model, we were able to intuitively track how individual samples undergo split decisions in the prediction process, thereby clarifying how the model makes sequential predictions based on specific feature values. This approach allows us to identify and quantify the relative contributions of each input feature to the model’s prediction outcome, thereby helping us understand which features play a critical role in classification decisions. **Figure 8** illustrates the feature importance and decision paths for DLR-LightGBM.

## 4 Discussion

Compared to previous approaches that relied solely on either radiomics or 2D deep learning, we propose a novel 2.5D deep learning model that integrates multi-slice MRI data to capture partial 3D spatial context while maintaining computational efficiency. Moreover, by integrating these features with clinical variables, we developed a nomogram model that demonstrated superior predictive performance. This multimodal fusion strategy, combined with interpretability analyses via Grad-CAM and tree-based visualization, enhances model transparency and offers new insights into prostate cancer aggressiveness assessment. The comprehensive model achieved an AUC of 0.957 on the training set and 0.919 on the testing set, outperforming any single model or combined radiomics and deep learning models. This indicates the joint model possesses strong generalizability and holds potential for clinical application in non-invasive prostate cancer aggressiveness assessment, particularly in screening and early intervention for high-risk patients.

Preoperative prostate MRI scanning plays a critical role in assessing prostate cancer and its aggressiveness. Although traditional multiparametric MRI (mpMRI) combined with the PI-RADS scoring system is widely used for diagnosis, its ability to grade cancer aggressiveness is limited and prone to interobserver variability. Further, prostate cancer typically appears as hypointense regions on T2-weighted imaging (T2WI), which offers high soft-tissue resolution, making it effective at distinguishing between benign and malignant lesions, as well as assessing tumor aggressiveness (14)​. However, due to the heterogeneity and irregular growth patterns of prostate lesions, manual lesion segmentation is often time-consuming and resource-intensive (15). Thus, the present study employed automated lesion segmentation based solely on the T2WI sequence. Unlike prior studies that segmented ROIs across multiple imaging sequences (16–18)​​, this study’s approach demonstrated high classification efficacy. This further reduced the need for manual intervention, simplified the training process, and validated the utility of T2WI in prostate cancer diagnosis. Compared with previous studies employing radiomic features or 2D deep learning models for prostate lesion classification, the present study innovatively integrated multilayer imaging data. Using a grid search, it identified the Inception_v3 neural network as the optimal method for feature extraction, constructing 2.5D deep learning features with partial 3D information (19). This method outperforms traditional 2D feature extraction by better capturing the spatial structure and heterogeneity of prostate cancer lesions, thereby enhancing model performance and predictive accuracy. However, due to the “black-box” nature of deep learning models, interpreting the biological significance of their features remains challenging (23). To address this, we employed Grad-CAM visualization (20), using activation maps to highlight key regions targeted during feature extraction. Among these 20 samples, 19 displayed heatmap activation areas that matched prostate cancer-diagnosed regions in pathology reports, with regions of high activation correlating to blurred or absent gland structures and abnormal cellular arrangement in pathology specimens. This result indicates that Grad-CAM-generated heatmaps effectively highlight key regions of model focus, aligning with actual lesion locations, and enhancing the model’s interpretability. This visualization analysis further validates the model’s effectiveness in identifying prostate cancer in imaging, thereby providing valuable insights for clinical diagnosis and future. In related research on prostate cancer aggressiveness by Cai et al., Grad-CAM visualization was applied to label-only prostate images, successfully localizing tumor regions (15). In the present study, radiomic features were extracted using the pyradiomics tool (version 3.0.1). Out of 1,834 radiomic features, 11 were selected, including one shape feature, two first-order statistics features, four GLCM, three GLSZM, and one GLRLM feature. The number of selected features suggests that GLCM features play a critical role in evaluating prostate cancer aggressiveness. GLCM describes the spatial relationships between pixels, providing detailed information on tumor texture, which can help to differentiate tissue complexity. Aggressive prostate cancers commonly exhibit more complex texture patterns, thereby indicating potential correlations with GLCM features (21).

In the present study, we developed Rad-LightGBM, DL-LightGBM, and DLR-LightGBM models, achieving AUCs of 0.758, 0.818, and 0.872 on the test set, respectively. Based on these results, we integrated the DLR-LightGBM output score (rad-score) with clinical features, including the PSA level, maximum tumor length, age, and PI-RADS score, to construct a combined model. The combined model, presented as a nomogram, achieved an AUC of 0.919 on the test set, significantly outperforming the individual models. This result indicates that integrating radiomics, 2.5D deep learning features, and clinical characteristics provides a more comprehensive representation of prostate cancer biology, thereby enhancing predictive accuracy. Compared with the study by Bertelli et al. (24), where a 2D deep learning and radiomic fusion model achieved an AUC of 0.875, our nomogram reached an AUC of 0.919, reflecting an enhanced predictive performance. Unlike Bertelli et al., our model integrates 2.5D features, enabling the capture of partial 3D spatial context while maintaining computational efficiency. Additionally, studies such as Prata et al. (23) and Chaddad et al. (22) reported lower AUCs (0.804 and 0.65, respectively) for similar tasks, highlighting the performance improvement our multimodal strategy offers. This multimodal feature fusion strategy holds potential for providing more clinically practical tools for prostate cancer aggressiveness assessment. Using DeLong’s test, the combined model demonstrated significant superiority over both the DLR-LightGBM and Clinic_LightGBM models in the training set (P<0.05) and near-significant superiority on the test set (P>0.05). In the test set, while the combined model still outperformed other models, its superiority was only near-significant (P>0.05), potentially due to the relatively small sample size of the test set. Nonetheless, the combined model achieved the highest AUC in the test set, thereby demonstrating robust generalizability to unseen data. Compared with other studies, such as those of Chaddad et al. (22), which reported an AUC of 0.65 for prostate aggressiveness prediction using T2WI and DWI, or Prata et al. (23), whose radiomic models based on T2WI and ADC achieved AUCs of 0.681 and 0.774, respectively, with 0.804 for the combined model incorporating clinical variables, our study demonstrated superior predictive performance. In the study by Bertelli et al. (24) using 2D deep learning and radiomics, the best-performing model achieved an AUC of 0.875. In our study, the combined model exhibited a superior predictive performance, thereby confirming the advantage of multimodal feature fusion in prostate cancer aggressiveness assessment. By integrating the 2.5D deep learning model with the T2WI sequence, this study provides a more precise and automated tool for assessing prostate cancer aggressiveness. This approach has the potential to improve preoperative evaluation accuracy, thereby assisting physicians in achieving more accurate individualized risk assessments and treatment decisions, ultimately enhancing patient outcomes. Our nomogram could be integrated into clinical workflows to enhance decision-making. For example, it could serve as a supplementary tool alongside PI-RADS scoring, helping radiologists prioritize high-risk lesions for biopsy. In a hypothetical clinical scenario, the model could be used to stratify patients into low- and high-risk groups, with the latter undergoing targeted biopsy or immediate intervention. This approach may reduce unnecessary biopsies and improve diagnostic efficiency.

Overall, the present study demonstrates the advantages of combining a 2.5D deep learning model with clinical features in assessing prostate cancer aggressiveness, though some limitations remain. First, this study used retrospective data, which may introduce sample selection bias, and the single-center design limits the model’s generalizability. The retrospective single-center nature of this study may introduce selection bias and reduce the generalizability of the model to broader populations. Overfitting could also occur due to the limited diversity of the training data. Future work will include external validation on multicenter cohorts to confirm robustness across varying clinical settings and imaging protocols. Future studies should be based on large-scale, multicenter datasets. Second, this study relied on manual lesion segmentation, with accuracy therefore being dependent on the operator’s experience and expertise. Variations among annotators may lead to inconsistencies in segmentation results. In future research, we plan to incorporate automated segmentation techniques to enhance the accuracy and efficiency of prostate cancer imaging analysis, to significantly reduce manual annotation time, and improve the standardization of image preprocessing.

## 5 Conclusion

Overall, in this study, we successfully developed an effective tool to evaluate prostate cancer aggressiveness by integrating prostate MRI with a 2.5D deep learning model, thereby significantly improving the accuracy and sensitivity of the assessment. Although certain limitations remain, this model’s potential for clinical application was preliminarily validated. Future efforts should focus on multicenter studies and integrating multimodal data to further optimize and expand the model’s applications, thereby providing stronger support for precision medicine and personalized treatment in prostate cancer.

## Data Availability

The raw data supporting the conclusions of this article will be made available by the authors, without undue reservation.
